# Making Modern Migraine Medieval: Men of Science, Hildegard of Bingen and the Life of a Retrospective Diagnosis

**DOI:** 10.1017/mdh.2014.28

**Published:** 2014-07

**Authors:** Katherine Foxhall

**Affiliations:** University of Leicester, School of History, University of Leicester, University Road, Leicester LE1 7RH, UK

**Keywords:** Charles Singer, Migraine, Hildegard of Bingen, Retrospective diagnosis, Medical imagery

## Abstract

Charles Singer’s retrospective diagnosis of Hildegard of Bingen as a migraine sufferer, first made in 1913, has become commonly accepted. This article uses Hildegard as a case study to shift our focus from a polarised debate about the merits or otherwise of retrospective diagnosis, to examine instead what happens when diagnoses take on lives of their own. It argues that simply championing or rejecting retrospective diagnosis is not enough; that we need instead to appreciate how, at the moment of creation, a diagnosis reflects the significance of particular medical signs and theories in historical context and how, when and why such diagnoses can come to do meaningful work when subsequently mobilised as scientific ‘fact’. This article first traces the emergence of a new formulation of migraine in the nineteenth century, then shows how this context enabled Singer to retrospectively diagnose Hildegard’s migraine and finally examines some of the ways in which this idea has gained popular and academic currency in the second half of the twentieth century. The case of Hildegard’s migraine reminds us of the need to historicise scientific evidence just as rigorously as we historicise our other material and it exposes the cumulative methodological problems that can occur when historians use science, and scientists use history on a casual basis.

## 1. Introduction

In 1913, a young scientist and historian named Charles Singer was in Germany researching precursors to modern theories of contagion. In Wiesbaden, he consulted the twelfth-century illuminated *Scivias* manuscript (c.1165) which described 26 religious visions experienced by the celebrated St Rupertsberg abbess, Hildegard of Bingen (1098–1179). In the stars, shimmering points of light and crenellated figures of some of the 35 illuminations in *Scivias*, Singer thought that he recognised depictions of ‘scintillating scotoma’. Noting that Hildegard had admitted to long periods of illness, Singer diagnosed a functional nervous disorder, specifically migraine.^1^ Nearly a century later, medical ideas about migraine have changed a great deal. Nevertheless, Singer’s retrospective diagnosis of Hildegard’s migraine has persisted, gaining popularity in the late twentieth century as the abbess’ reputation has grown. Hildegard’s migraine has appeared in some significant places, including Oliver Sacks’ *Migraine* (1970), scholarly publications in medieval history and a pharmaceutical marketing campaign. More recently, on the internet, contributors to blogs and websites have freely proposed Hildegard as a ‘patron saint’ of migraine and migraineurs.

There is a large scholarly output in English, German and French by historians specialising in Hildegardian studies. This grapples (among other themes) with whether Hildegard suffered from migraine, whether she was the designer of the *Scivias* illuminations, and whether she was the author of the medical text *Causae et Curae*. I should make it clear from the outset that while I am not attempting to make new claims about Hildegard’s own experiences of illness or the authenticity of the works that have been attributed to her *oeuvre*, this literature has played an important part in informing this piece; each of these three questions has their own independent scholarly history, but they converged in Singer’s argument.^2^

Instead, I am interested here in tracing a twentieth-century story about how the creation of new medical understandings of migraine provided Charles Singer with evidence to diagnose Hildegard’s migraine and how, subsequently, this diagnosis has been used in different ways to help authenticate particular arguments about illness in the past and present. In doing so, I do not aim to reject retrospective diagnosis *tout court*. Much of the extant debate on retrospective diagnosis has revolved around the motives of those making the diagnosis, or has traced how historical figures have attracted competing diagnoses as medical knowledge has changed. Here, I wish to use Hildegard as a case study that shifts our focus from a polarised debate about the merits or otherwise of retrospective diagnosis as an act, and to examine instead what happens when diagnoses take on lives of their own. Simply championing or rejecting retrospective diagnosis is not enough. I argue that we need instead to appreciate not only how, at the moment of creation, a diagnosis reflects the significance of particular medical signs and theories in any given historical context but also how, when and why such diagnoses can come to do subsequent meaningful work when mobilised as scientific ‘fact’.

The first section of this article charts a pre-history to Singer’s diagnosis, in the emergence of a new dominant medical formulation of migraine in the nineteenth century, which emphasised migraine as a disorder associated with aura, vision and science. The second section examines how Singer used this formulation to identify Hildegard’s migraine, and how in turn this advanced his own arguments about the medieval development of science. Third, I look at how Hildegard’s migraine took on new life in the second half of the twentieth century in Oliver Sacks’ incorporation of Singer’s ideas and Hildegard’s imagery in his famous study *Migraine*, and the endeavours of historians to assert the authenticity of Hildegard’s manuscripts, using the migraine diagnosis as evidence. In each case, I show how Hildegard’s migraine has proved attractive not because of its ‘truth’, but because it served to advance a particular argument or position. In the final section, I demonstrate the methodological problems that can result from the decontextualisation and accumulation over time of what Felicity Callard has termed ‘interdisciplinary borrowings’. The case of Hildegard’s migraine reminds us of the need to historicise scientific evidence just as rigorously as we historicise our images and texts.^3^

## 2. Retrospective Diagnosis

The issue of retrospective diagnosis has long exercised historians of medicine. Do histories that mine the past for symptoms or traces corresponding to current medical knowledge tell us anything about medical history? Or should we write histories that respect and contextualise historical knowledge on its own terms? This approach accepts, in Andrew Cunningham’s words, that ‘you die of what your doctor says you die of’, however outdated these contemporary pronouncements might now seem to our modern gaze.^4^ While practitioners of retrospective diagnosis consider the employment of modern biomedical categories as a perfectly valid way to talk about disease in the past, others have criticised the practice’s tendency to reduce the lives of individuals to ‘the mere expression of disease’. Roger Cooter, for example, has dismissed retrospective diagnosis as ‘inherently condescending’.^5^ The discussion about how to approach histories of disease and illness has re-emerged with renewed vigour in recent years with the publication of a slew of disease ‘biographies’.^6^

We might usefully think of retrospective diagnosis practice as a broad spectrum, whose ends bear little similarity to each other. At one end of the scale well-funded paleopathology research groups utilise expensive scientific technology to identify pathogens in archaeological remains.^7^ High-profile examples of this include the 1976 examination of the mummy of Ramses II by French scientists who wished to prove that the Egyptian pharaoh died from tuberculosis.^8^ The question of whether or not the pathogen *Yersinia pestis* was responsible for the ravages of the Black Death in the medieval and early-modern period almost constitutes a sub-discipline.^9^ Recently, Monica Green has emphasised the value of cross-disciplinary collaborations that take the biological materiality of disease seriously on both global and deep chronological scales. Combining scientific disciplines including paleopathology and genetics with professional history offers a very different kind of disease work than the ‘parlour game’ that imposes various illnesses on historical figures through the interpretation of texts, artefacts, images and commentary.^10^ Was King George III’s madness actually porphyria? Did Nietzsche have syphilis? These kinds of diagnoses have widely been criticised; Caroline Walker Bynum’s discussion in *Holy Feast and Holy Fast* (1987) regarding whether medieval women can be said to have had ‘anorexia’ remains one of the most convincing explorations of the problems and implications of making such a diagnosis if our aim is to understand a period historically. In particular, Bynum criticised a tendency to assume that we can identify either a ‘secularisation’ or ‘medicalisation’ of behaviour previously regarded as religious, noting that a number of medieval paradigms existed for ‘not eating’. She also noted that the validity of a diagnosis depends on which modern definition of an illness is chosen.^11^ If we take examples of retrospective diagnoses as historical artefacts in themselves, however, they can be very revealing. In a recent survey of theories about Jonathan Swift’s ailments over three centuries, for example, Marjorie Lorch notes that while the evidence regarding Swift is inadequate to determine the accuracy of any individual retrospective diagnosis of his ailments, analysing how these diagnoses have changed reveals something of how ‘different signs and symptoms were given status and significance by different writers at different historical periods’.^12^

Migraine leaves no physical trace, and offers no scope for bioarchaeological analysis: the idea of Hildegard’s migraine derives from one man’s interpretation of imagery and words. Hildegard’s case is important because Singer’s diagnosis has persisted beyond the arena of medical hypothesising, and taken on a life of its own as historical evidence in a number of different ways over a long period of time.^13^ So can thinking historically about Singer’s diagnosis tell us anything? For a start, it is worth noting that people in the medieval period certainly did have knowledge of, and treatments for, a disorder commonly known as *emigranea*, a corruption of Galen’s second-century term *hemicrania* which remains the root of the modern term ‘migraine’. The text of *Causae et Curae*, generally accepted by recent scholars (contrary to Singer’s view, as we will see below) to be Hildegard of Bingen’s work, albeit a rough draft that she never managed to put into final form, explained *emigranea* in classical terms, as a disorder that stemmed from melancholy (black bile), and ‘all bad humours present in a person’.^14^ Migraine seizes only half the brain at a time because ‘its strength is such that if it seized the whole head, a person would not be able to endure it’. Treatment involved reducing aloe and myrrh to a fine powder, mixing with wheat flour and poppy oil to make a dough, then covering the whole head. The patient should place a cap over the top and keep on the head for three days and nights. The treatment would sedate the pain and enrich the brain.^15^ Another recipe from eleventh-century Chartres recommended the use of peony root frequently stroked over the site of pain, taking a bath with sweet smelling herbs boiled in vinegar or using a cap made with well-boiled hot abrotano (now known as Artemisia).^16^ A thirteenth-century Welsh text recommends a patient to eat a baked or roasted hare’s brain stuffed with rosemary flowers, followed by sleep to treat the ‘migran’.^17^ Emigranea was thus understood as a very different disorder to the illness that Hildegard described as her own personal suffering; the best recent translation describes ‘a clouding over of my eyes, and I was so pressed down by the weight of my body that I could not raise myself. So I lay there overwhelmed by my intense pains’. Later she described ‘excruciating airs’ coursing through her whole body, and the ‘marrow in my bones dried up so much it was as if my soul must be released from the body’.^18^

It is almost certain that Hildegard would have made no connection between these pains and those of *emigranea*, not least because in this instance she interpreted her illness as divine punishment. By 1917, however, when Charles Singer diagnosed Hildegard with migraine, a very different idea of migraine had emerged that did correspond to some extent with Hildegard’s account. Singer’s retrospective diagnosis endowed Hildegard with a migraine devoid of humours, and defined instead by the neurological experience of disordered vision; this was an understanding that had only existed since the 1870s. Singer made his argument based on apparent imagery of migraine aura that he found in the illustrations of the twelfth-century *Scivias* manuscript, using the texts in which Hildegard described her experience of illness in support.

## 3. Singer and Hildegard

Charles Singer (1876–1960) is best known as the first president of the British Society for the History of Science, founded in 1947. After studying biology, zoology and medicine, Singer chose the latter for his early career, working in Abyssinia, Singapore and Sussex before being appointed as medical officer to the London Cancer Hospital in 1909.^19^ Following his marriage to the medieval historian Dorothy Waley Cohen in 1910, he became increasingly interested in history. His first published articles, on germ theory in Benjamin Marten’s *New Theory of Consumptions* (1720), early references to tropical diseases and the history of tobacco were received well by his historically inclined peers. Charles Ryell, surgeon to the London Cancer Hospital, thought Singer’s unearthing of Marten’s ‘bygone and forgotten forerunner’ of germ theory to be ‘very enterprising’. Fielding H. Garrison received Singer’s next articles on ‘Early References to Tropical Diseases’ and the history of tobacco with similar approval: Garrison reported ‘a great deal of interest’ among the officers of the Army Medical School, who felt that the history of medicine was ‘as yet formless and void.’^20^

In 1912, Charles and Dorothy moved to Heidelberg, where he continued his search for predecessors to modern medical concerns and ideas. The librarian at Wiesbaden allowed Charles to consult the illuminated manuscript of *Scivias*. This was Hildegard of Bingen’s record of her religious visions. From an early age she had experienced waking visions, and a light she described as ‘so great a light that her soul trembled’. In her early forties, ‘the great pressure of my pains’ propelled Hildegard to explain these visions.^21^ Through long periods of illness, Hildegard worked for a decade on the text that would become *Scivias*. In 1148, she received papal approval to write theological works, and subsequently attracted widespread fame as a visionary, preacher and reformer.^22^ *Scivias* was completed in 1151; it is at once a work of orthodox theology and a remarkable record of Hildegard’s prophetic visions.^23^

Since around 1979, the eight-hundredth anniversary of Hildegard’s death, and 1998, when a number of publications commemorated the nine-hundredth year since her birth, Hildegard has attracted sustained attention both by academics, and in popular culture. Her life has been the subject of a feature film, and in Germany a system of alternative holistic healing bears her name.^24^ In May 2012, Pope Benedict XVI formally canonised Hildegard before in October of the same year proclaiming her a ‘Doctor of the Church’ in recognition of her teachings, only the fourth woman to receive the honour. Hildegard would have been, Barbara Newman has suggested, ‘extraordinary in any age’, but for a woman of the twelfth century, her ‘achievements baffle thought’.^25^ In addition to recognising the significance of her theological works, historians have granted Hildegard a role in medieval ideas about gender, the human body and nature. Her oeuvre also includes over seventy liturgical compositions, and a religious drama. Increasingly, Hildegard’s scholars have focused on her medical, as well as religious, ideas.^26^ Victoria Sweet, for example, has seen Hildegard as an ‘informant’, who helps us to understand the tenacity of a pre-modern medical model of elements, qualities and humours.^27^

**Figure 1:**
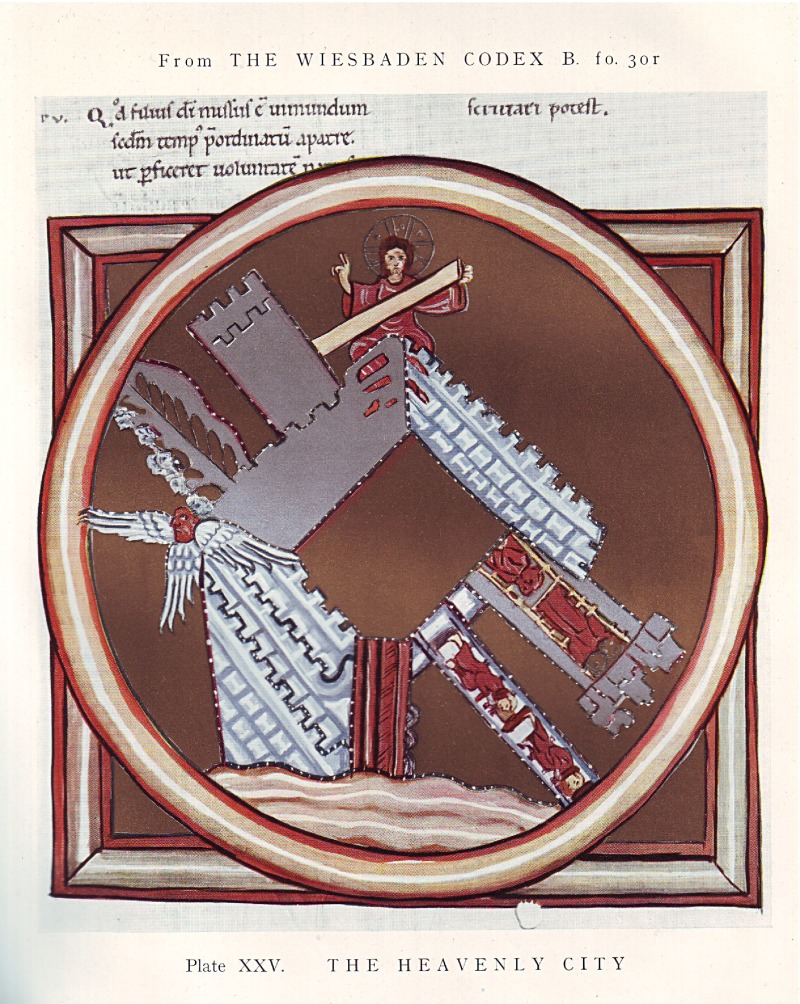
(Colour online) ‘The Heavenly City’, miniature from *Scivias* (c.1165), reproduced in Charles Singer (ed.) *Studies in the History and Method of Science* (Oxford, 1917).

If modern scholars have begun to analyse Hildegard’s medicine on its own terms, Singer’s interest in the second decade of the twentieth century lay in retrospectively using modern medical categories to place Hildegard in a long history of scientific progress. Singer’s attention to Hildegard reflected contemporary historical and antiquarian endeavours to discover, collect, translate and re-publish a plethora of medieval texts, including those attributed to Hildegard. In 1900, Melina Lepinska had described *Scivias* as ‘one of the most remarkable of Hildegard’s works’.^28^ On seeing the extraordinary Wiesbaden manuscript of *Scivias* for himself, Singer abandoned his work on contagion, and turned his attention to the stars, crenellated shapes, shining lights, fortification figures and concentric circles that characterised the miniatures of Hildegard of Bingen’s religious visions. Singer ‘recognised at once that the figures (…) resembled descriptions by patients of what they had seen during attacks of migraine’ (Figure 1).^29^ His private letters reveal that he initially shared his ideas with the Swiss physician and historian Arnold Klebs. ‘I was very glad to have the chance to see those beautiful reproductions of the Hildegard manuscripts’, Klebs wrote in the summer of 1913,

and the more I think about it the more I become convinced that you have discovered an eminently interesting subject (…) I do not think anyone in England is tackling medical history with such thoroughness and energy as you do and I have no doubt that in a few years you will have become so expert that we will have to flock to London to learn from you.

Three weeks later, Klebs wrote again:

Do not forget that I am anxious to get your Hildegard photographs. I should like to get an *illuminated* set. I should very much like to show them in America, naturally as coming from you, with your theories on the subject.^30^

Perhaps gaining confidence from Klebs’ enthusiasm, Singer presented his ideas about Hildegard in public before the Historical Section of the Royal Society of Medicine in November 1913. In his talk, he showed the photographs and coloured reproductions of the manuscript illuminations. One member of the audience, Dr R. Hingston Fox, spoke as a personal sufferer from migraine, and agreed that Singer had proved his case. He also suggested to Singer that ‘the blue colours in the pictures were as important as the red, both these hues, as well as others, being characteristic of migrainous spectra’.^31^ William Osler, Regius Professor of Medicine at Oxford, urged Singer to publish his research, and in 1914 invited Singer to take up the Philip Walker Studentship in Pathology at Oxford University. From then on, Singer was enabled to devote virtually all of his time to the history of medicine and science and in June 1914 Dorothea and Charles returned to Wiesbaden to consult the *Scivias* manuscript again. By late July, travellers’ cheques ceased to be payable in German currency as war with France threatened. The Singers left for Holland, undertaking ‘a most trying journey across the line of German mobilisation (…) we gathered a small party of English and Americans. All of us were nearly penniless and had been forced to abandon our luggage’. Singer’s bags had contained the manuscript of his Hildegard essay. So, after he arrived back in England, Singer rewrote the article from memory. ‘I rather think I improved it’, he later commented.^32^

Singer’s re-written article, ‘The Scientific views and visions of Saint Hildegard (1098–1880)’, was finally published in full in 1917, as the first chapter of his edited collection *Studies in the History and Method of Science*. His theory about the migrainous pathological basis for Hildegard’s religious visions constituted only a four-page coda to a fifty-five-page chapter devoted in the main to explaining Hildegard’s ideas on scientific subjects including the structure of the universe, microcosm and macrocosm, anatomy, physiology, birth, death and the soul. He noted that Hildegard had suffered ill health for much of her life and drew further evidence from Hildegard’s writings about her religious visions, which she experienced ‘neither in sleep, nor in dream, nor in madness (….) But wakeful, alert’.^33^ It seemed clear to Singer that Hildegard’s repeated complete recoveries, activity between attacks and long life indicated a functional nervous disorder, an unwieldy category which at this time encompassed neurasthenia, neuralgia and hysteria. Singer diagnosed migraine. ‘In the “more typical” of her visions’, he wrote, ‘the medical reader or the sufferer from migraine will, we think, easily recognise the symptoms of scintillating scotoma’. To understand where Singer’s ideas about migraine came from, and his references to patients’ descriptions of visual aura we must return to the discussions of scientific men in the middle of the nineteenth century.

## 4. Migraine and Aura

On 30 September 1858, Sir John Herschel (1792–1871), mathematician, astronomer, chemist and photographer, gave a lecture on ‘Sensorial Vision’ to the gathered members of the Leeds Philosophical Society. He told how one morning, at his breakfast table, he had watched a ‘singular shadowy appearance’ at the outside corner of his left field of vision. The pattern appeared ‘in straight-lined angular forms, very much in general aspect like the drawing of a fortification, with salient and re-entering angles, bastions and ravelins, with some suspicion of faint lines of colour between the dark lines’.^34^ Herschel was not alone in publicly recording such a personal experience. In 1865, David Brewster, natural philosopher and inventor of the kaleidoscope, discussed his experiences of ocular spectra, this time in the *Philosophical Magazine* as a way to contribute to theories about the structure of the optic nerve. Later the same year, the Astronomer Royal, George Biddell Airy, also published an account of his periodic attacks of hemiopsy. In these papers, these men of science did not tend to dwell on other forms of pain or suffering associated with their visual disturbance. Brewster, for example, noted that his attacks ‘were never accompanied either with headache or gastric disturbance’ while Airy, too, observed that ‘in general, I feel no further inconvenience from it’, although his friends found their attacks ‘followed by oppressive head-ache’.^35^ These men were less interested in illness *per se* than they were in using their personal experiences to advance discussions about optics, vision and light that got to the heart of their beliefs about scientific authority, and indeed the very possibilities of seeing accurately and objectively with the naked eye. Elizabeth Green Musselman has written in detail about the ill health suffered by nineteenth-century men and women of science. She argues that they found meaning in nervous illnesses and failings such as hemiopsy, colour blindness and hallucinations by applying their ideas about refined, well-managed, efficient nervous systems to science and society in general.^36^ While these discussions about ‘half-blindness’ contributed to contemporary scientific questions, their public airing also contributed to making these transitory visual attacks socially acceptable, even desirable.

**Figure 2:**
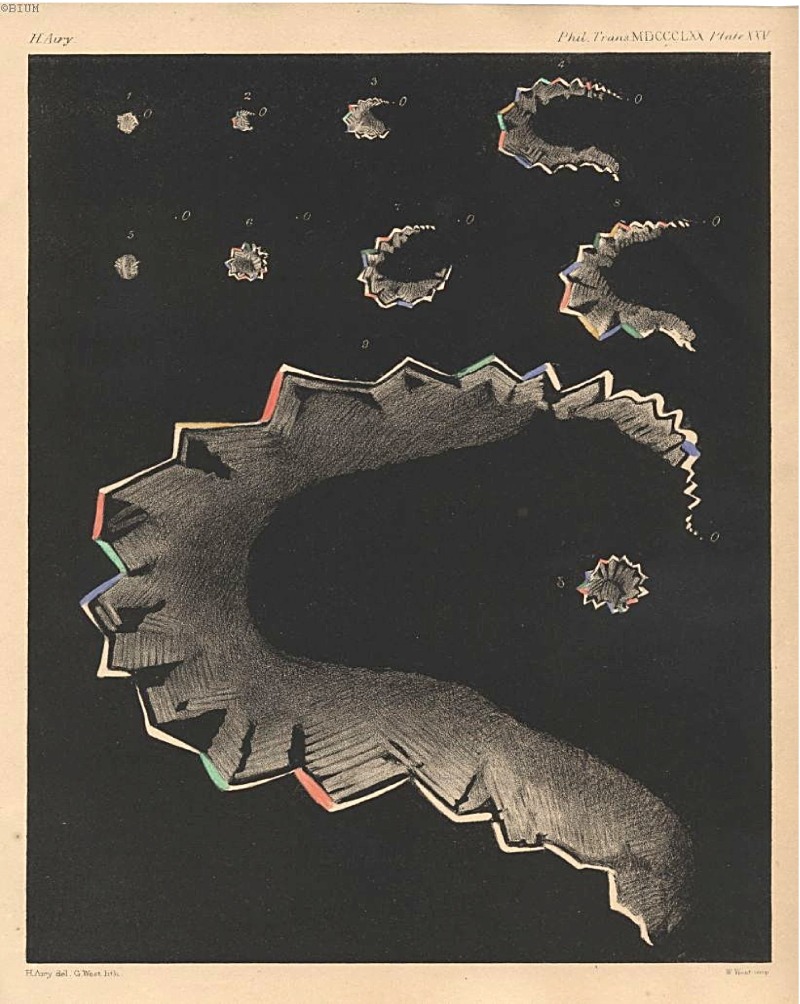
(Colour online) Plate XXV ‘Stages of Teichopsia’ from Hubert Airy, ‘On a Distinct Form of Transient Hemiopsia’, *Philosophical Transactions of the Royal Society of London*, 160 (1870).

In a subsequent essay in the *Philosophical Transactions of the Royal Society of London* (1870), Hubert Airy, the physician son of the Astronomer Royal, drew together these accounts to propose that all of them (including himself) had experienced a phenomenon that he termed ‘transient teichopsia’. Hubert did admit to suffering from terrible headache after the ‘blindness’, but did not consider that these visual phenomena were ‘merely’ a disease: such disorders would be ‘hardly deserving of the attention of scientific men’. Rather, these functional disturbances should be regarded as ‘a veritable “Photograph” of a morbid process going on in the brain’, in which case, he thought, ‘their interest and importance cannot be too strongly insisted upon’.^37^ Airy accompanied his paper with arguably some of the most beautiful imagery in the history of medicine: a series of sketches illustrating his own experiences of the disturbance across his visual field (Figure 2). With ‘changing gleams’ of red, blue, yellow, green and orange, at its height the vision ‘seemed like a fortified town with bastions all round it’. The whole experience lasted half an hour.^38^ Despite the variety of words these men employed to discuss hemiopsy, it is important to emphasise that they made virtually no association between their discussions and any of the contemporary terms for migraine (including *megrim*, *hemicrania*, sick, nervous or bilious headache).

A young Cambridge physician named Edward Liveing was in the audience for Airy’s presentation of his paper to the Royal Society. He was impressed by Airy’s careful observation and minute descriptions, as well as his ‘excellent’ drawings of the spectral appearances. For several years, Liveing had been collecting his own information and patient case notes about a group of ailments including sick, blind and bilious headaches, as well as hemicrania and hemiopsy (Liveing used the term ‘hemiopia’). Liveing believed that these were all closely related. This was not an entirely new argument, but Liveing felt that English physicians needed to better understand these disorders as a family in order to catch up with the more comprehensive knowledge enjoyed by their French and German peers. Liveing brought these disorders under the umbrella term of *megrim*, which he explained was part of a larger family of functional disorders that included epilepsy, asthma and angina pectoris, all of which were characterised by paroxysms or fits. Liveing’s book *On Megrim, Sick Headache and Some Allied Disorders* (1873) is now often considered to be a founding contribution to modern understandings of migraine in the English language. Memorably, Liveing proposed that attacks of *megrim* in all their manifestations were the result of an event in the body analogous to ‘nerve-storm’: a periodic dispersion of accumulated nervous energy.^39^

In the same year, another Cambridge physician, Peter W. Latham, published a second short book of two lectures *On Nervous or Sick-Headache*. In contrast to Liveing’s nerve-storm theory, Latham explained the cause of migraine’s visual aura as a contraction of the blood vessels of the brain, diminishing the blood supply and disturbing vision, followed by the vessels’ ‘dilatation’ to bring on headache.^40^ Despite their theoretical differences, both books dedicated substantial space to reprinting the discussions that men of science had been having about visual disorders. In so doing, Liveing and Latham took migraine from the largely ignored realms of domestic recipe books, patent remedies and the classified pages of newspapers and infused it with a new authority, relevance and *cachet* through a firm connection with vision. These discussions were highly gendered and classed. ‘Perhaps in an University town’, Latham commented, ‘it may be more prevalent among males than in other places’. Liveing also explained the disorder as a result of strained intellectual faculties, attacking over-worked students, ‘literary men’ or men who entered ‘the more serious business of life’. Women were not exempt, but by contrast to men, Liveing’s kind of megrim seemed to attack seamstresses or ‘poor women exhausted from over-suckling’.^41^ Throughout the century, medical men had identified lower-class working women, their bodies and nerves broken down by exhaustion, overwork and poor diet, as the main sufferers of migrainous headaches, but megrim’s emerging social visibility and acceptability in the late nineteenth century derived in large part from the central role that new theories about nerves gave to the personal testimony of scientific men, not to the experiences of seamstresses and nurses.^42^ In the remaining decades of the nineteenth century physicians from Britain and beyond continued to elaborate their own experiences of migraine in medical journals on both sides of the Atlantic.^43^

The most important aspect of these discussions for understanding the background to Singer’s diagnosis of Hildegard’s migraine is that Liveing and Latham also both prominently reproduced Hubert Airy’s diagrams in their studies. Liveing included the image as a double-page pull-out, in full colour, between the index and an analytical table of cases at the end of *On Megrim*. Latham reproduced it in black and white as the frontispiece to his book. By using Airy’s image in key position in their books, the two physicians transformed Airy’s undeniably beautiful, and instantly recognisable, analysis of his personal experience into a standard bearer for the authentic representation of migraine experience, irrespective of theoretical standpoint. ‘[F]rom an occasional and relatively unimportant curiosity’, Mervyn Eadie has observed, migraine with visual aura had become ‘a significant clinical entity in the medicine of English-speaking countries’.^44^ In 1895, Sir William Gowers gave the prestigious Bowman lecture to the British Ophthalmological Society in London on the topic of ‘Subjective Visual Sensations’. He commented on the ‘elaborateness and precision’ of Airy’s drawings. Because Airy made notes at the time, and drew his observations ‘with purposed care’, Gowers declared that they held ‘very great weight’ as scientific records.^45^ Thus, by the end of the nineteenth century, Airy’s images had become an example of what Lorraine Daston and Peter Galison have termed a ‘working object’ for migraine: a standardised exemplar that taught practitioners ‘what is worth looking at, how it looks and perhaps most importantly how it should be looked at’.^46^

When, two decades later, Charles Singer diagnosed Hildegard’s migraine by comparing the manuscript images in *Scivias* to descriptions produced by migraine patients, he could only have been thinking of these famous, oft-reproduced (Airy’s image was even reproduced by Jean-Martin Charcot) and instantly recognisable migraine motifs: although other images of migraine had existed, they had been dismissed, and fallen to the wayside in this new formulation of migraine as a disorder experienced by eminent ‘men of science’, and epitomised by Airy’s image.^47^ Bolstering Singer’s apparently straightforward recognition of migraine aura in Hildegard’s illuminations, therefore, was a very specific and recent articulation of migraine as a highly gendered disorder associated most strongly with genius, scientific vision and the intensity of male intellectual work. Singer’s diagnosis retrospectively endowed the abbess with a disorder that had become accepted in late-Victorian expectations of the physical and mental constitution of a ‘scientist’. Using his medical knowledge to render the unusual patterns in Hildegard’s religious imagery as the manifestations of a neurological disorder was important because it enabled Singer to sideline Hildegard’s theology and replace it with ‘science’ as the basis for her philosophy of the world. Then, Singer could fit Hildegard into his larger story of scientific progress from the superstitious darkness of the Middle Ages to the light and reason of modern science. Singer was by no means the only scholar creating medieval ‘scientists’ through such a secularising treatment. Amanda Power has explored how late nineteenth-century scholars also resurrected the reputation of Roger Bacon, for example, by presenting him ‘not so much as a man, but as a “stage” in a long, triumphant [scientific] history’ which ‘edited out’ the Christian faith intrinsic to Bacon’s work.^48^

From his earliest forays into historical research, Singer had been attempting to formulate a meaningful role for the teaching of history for understanding the very nature of medicine and science. In 1913, Singer had likened the progress of scientific knowledge ‘to the erection of a great building in which no part of the general structure can go far without due attention being given to the other parts’.^49^ As Anna K. Mayer has argued, in the aftermath of the First World War Singer would come to see the history of science as an international exercise in scientific humanism, the value of which ‘lay in addressing an age that experimented both with rampant scientific and technological progress and with democracy’. Singer was of a generation of science historians whose knowledge of experimental science was crucial for establishing their vision of history, and their belief in their authority to impart that vision.^50^ For Singer, Hildegard was a key piece in this puzzle. Compared with the ‘dark degradation’ of her contemporaries, he argued that Hildegard was ‘feeling her way to rational explanation of the world’. His twentieth-century readers needed simply ‘to get past the bizarre and visionary form’ into which she cast her theory of the essential similarity of macrocosm and microcosm to find a ‘systematic and skilful presentment’ of a scientific philosophy characterised not by religion, but by common sense, which gave meaning to the facts of nature.^51^ Singer had definite views about which of her attributed works Hildegard was actually responsible for. For example, he began his article by rejecting recent claims (that he had elsewhere previously dismissed as a ‘large and tedious literature’) that she was the author of the work known as *Causae et Curae*. Singer believed that the manuscript of *Causae et Curae* was ‘ill-written’ and its association with Hildegard was therefore ‘spurious’. ‘Nothing could be more unlike the ecstatic but well-ordered and systematic work of the phrophetess of Bingen than the prosy disorder’ of the ‘scrappy paragraphs’ of *Causae et Curae*, he wrote. Singer knew that this argument put him in the minority at the time, but the position was crucial for his own construction of Hildegard’s persona; although he agreed that the content of *Causae et Curae* was definitely scientific ‘in character’, admitting her authorship would detract from the high quality of the science to be found (paradoxically) in her theological works, particularly *Scivias*.^52^ It is also distinctly possible that Singer recognised that *Causae et Curae* would undermine his argument in a second way, by providing a medieval definition of migraine that bore no relation to the illnesses Hildegard had experienced.

In dealing with Hildegard, Singer thus made three manoeuvres: he diagnosed Hildegard’s religious visions as being migrainous in origin; he sidelined her religion; and he rejected her authorship of ‘unscientific’ works. Together, these moves all contributed to Singer’s representation of Hildegard as a scientist. In the years he had been working on Hildegard, Singer had developed a firm belief in the early chronology of scientific development, and in science itself as the ‘making of knowledge’. The Middle Ages had witnessed slow decline from ‘the intellectual efficiency of classical antiquity’, reaching ‘the point of lowest degradation of the human intellect’ in the tenth century. From then on, science enjoyed a slow ascent, before a considerable extension of natural knowledge in the thirteenth and fourteenth centuries. Hildegard advanced a coherent philosophy at a critical point in this chronology. Singer insisted on the need to approach history scientifically, to ‘interpret the past in the light of present knowledge’, which was exactly what he had done in diagnosing Hildegard’s migraine.^53^

Singer published a second version of the Hildegard essay in 1928, this time in a collection of essays that laid out his own fully formed theory of the history of scientific progress. *From Magic to Science* represented his attempt to trace the collapse of ancient science ‘into the swamp of magic’ and the first attempts to recover ‘from that hideous slough’. Singer reiterated that Hildegard’s work was ‘in fact, Science, and with her we have left the Dark Ages and the Dawn has begun’. He acknowledged that his original essay on Hildegard had aroused criticism from his contemporaries but noted that their complaints had focused on disagreeing with his rejection of Hildegard’s authorship of *Causae et Curae*, not on his application of medical theory to account for ‘the pathological basis’ of the visions in *Scivias*. This had ‘been generally accepted by those with any knowledge’ of migraine. He all but cut the contested section about *Causae et Curae* from the revised article (a point to which we will return later), but reinforced the argument about migraine; the 1928 version of the article included new captions for the coloured plates which pointed out the migrainous features of the imagery to the lay reader.^54^

Diagnosing migraine provided a way for Singer to make Hildegard into a scientist, but in the years following others identified with her as a sufferer. In 1932, for example, R.H. Elliott reported that when he had shown Hildegard of Bingen’s illustrations to several of his patients in consultations, they had recognised features of their own migraine attacks. Demonstrating a more positive view of religion than Singer, Elliott noted that a ‘richness’ of migraine symptoms was ‘more often observed in clever intellectual people endowed with the creative type of mind’, and went on to diagnose Moses, Jeremiah, Ezekiel and Daniel, Paul of Tarsus, St John the Divine and Zoroaster as migraine sufferers.^55^ Elliott’s casual diagnosis of a swathe of biblical and religious figures as migraineurs might seem far-fetched, but it illuminates two points. First, it shows the extent to which the idea of migraine as a disorder associated with creativity, intellect and visual disturbance had gained ground since Liveing’s work in the 1870s. In this formulation other kinds of migraine had receded from view, particularly the older vernacular meanings implied by the word *megrim*, which incorporated the key symptoms of sickness and headache. Second, Elliott’s discussion of his patients reinforced Dr Hingston Fox’s reaction in 1913 to Singer’s original proposal that Hildegard suffered from migraine: these apparent representations of illness experience seemed to speak across the centuries: history became part of Elliott’s clinical encounter. Beyond Singer’s schema for the history of science, Hildegard had become a sufferer of a timeless disorder.

## 5. Oliver Sacks

Notwithstanding Elliott’s paper, however, and despite being reprinted in 1928 and 1958, Singer’s ideas about Hildegard’s migraine attracted relatively little attention during the first half of the twentieth century. Physicians interested in functional nervous disorders turned to examining the physical and emotional effects of the 1914–18 war, and ideas about migraine fragmented as researchers suggested roles for pituitary swelling, brain swelling, allergies, endocrine organs and psychological factors. In 1933, Critchley and Ferguson summed up the lack of direction; migraine had become ‘the happy hunting ground of the theorist (…) attacked by representatives of all branches of medicine’.^56^ From the 1940s, the influence of the American neurologist Harold Wolff revived vascular theories as a dominant explanation for migraine mechanisms. In the late 1960s, a young neurologist, Oliver Sacks, was inspired to write a new book on migraine after working in a migraine clinic and reading Liveing’s *On Megrim*.^57^ Sacks had also been entirely convinced by Singer’s argument, agreeing that the *Scivias* images were ‘indisputably migrainous’. Sacks went further than Singer, and reduced the abbess’ allegorical interpretation of her ecstatic inspiration entirely to physiological process: Hildegard had experienced ‘a shower of phosphenes in transit across the visual field, their passage being succeeded by a negative scotoma’.^58^

Sacks’ book, published in 1970, was designed for a general readership and quickly became a seminal account of the history, experience and state of medical knowledge about migraine. Through many editions, revised and expanded in 1985 and again in 1990, it has attracted millions of readers since its first publication. There are two ways to understand how Hildegard performed an important function in Sacks’ work. First, is the treatment of Hildegard’s migraine in the text. In a similar way to how Singer had organised his article, Sacks initially discussed Hildegard’s migraine almost as a coda to the main scientific discussion around migraine aura. Sacks made no particularly grand historical claim for the importance of Hildegard’s migraine; relegated to an appendix, at first glance the section seems little more than of incidental interest. However, the discussion of Hildegard was placed at the end of Sacks’ ‘largest, strangest’ chapter, in which he argued that although aura lay at the very heart of migraine, nobody had given it sufficient attention since Liveing.^59^ Sacks’ emphasis on migraine aura in 1970 was significant; during the 1950s and 1960s Harold Wolff’s experimental research had firmly established a vascular theory of cranial vessel dilation as the primary explanation for the pathogenesis of migraine.^60^ Sacks did not dismiss the importance of vascular changes as explaining the cause of migraine headaches, but argued that they did ‘nothing to explain the origin of migraine *attacks*’.^61^ Sacks’ championing of Hildegard’s migraine thus used history to reinforce a disciplinary statement about the primacy of neurological models for understanding migraine, a question that continues to provoke hostile exchanges today.^62^

Sacks presented Hildegard’s migraine in a second way: a black and white line drawing based on the *Scivias* miniature ‘Vision of the Heavenly City’ has provided the book’s frontispiece for every edition.^63^ The permanency of this image is a striking contrast to the constantly updated images and changing titles that have graced *Migraine’s* covers.^64^ The caption accompanying the frontispiece unambiguously tells readers that the figure is ‘a reconstruction from several versions of migrainous origin’. The text within the book regarding Hildegard has also remained unchanged through the revisions and updates to *Migraine*. The preface to the 1992 edition opens by stating that

the chief features of migraine – its phenomena, and how these are experienced by the patient, its mode of occurrence, the triggers that may provoke it, the general ways in which one may live with it or combat it – none of these has changed in 2000 years.^65^

Such a bold statement needs anchoring and Hildegard implicitly acts as such a grounding figure. Her unchanging migraine legitimates Singer’s neurological medical model for migraine in two millennia of history.

At this point, Sally Shuttleworth’s analysis of the growth of child psychology in the late nineteenth century provides a useful parallel. Shuttleworth recounts the example of an insane baby with enough strength to require restraint by four people, first reported in 1746. In the late nineteenth century a number of medical commentators including the influential psychiatrists James Crichton Browne and Henry Maudsley cited the story with ‘utter credulity’ to insist that children could suffer from insanity. Shuttleworth argues that medical writers have often used such ‘clinical legends’ anachronistically and unproblematically for their own ends.^66^ Once transformed into ‘cases’ by scientific authority, such historical stories take on textual lives of their own as they are repeated over decades. Sacks’ use of Hildegard’s migraine as history is a good example of Shuttleworth’s argument but it also illustrates the paradox that such cases gain authority precisely by becoming increasingly detached from the disciplinary contexts and burdens of proof that supported their elucidation in the first place.

## 6. Medieval Historians and Hildegard’s Migraine

I want to stay with this question of contextual detachment, because seemingly far removed from medical debates about neural or vascular theories, the question of Hildegard’s migraine has appeared in discussions that get to the heart of medieval historiographical methodology. For some historians, the authority of a ‘medical’ diagnosis has established Hildegard’s migraine as common knowledge and clinical fact. For example, in her 2010 study of pain in medieval culture, Esther Cohen notes that ‘when Hildegard of Bingen’s will was crossed (…) she could take to her bed with violent migraines and write about them as eloquently as any man to the abbots or archbishops who had rejected her liturgy’. It is striking that while Cohen carefully deconstructs Hildegard’s illness accounts as a tool in her dealings with the Catholic Church, Singer’s diagnosis requires no qualification or unpicking, even though the actual letter to which Cohen refers describes only ‘a grievous illness’.^67^

Other medieval historians have engaged directly with the diagnosis. In 1985 the historian Barbara Newman dismissed the relevance of Charles Singer’s hypothesis for understanding Hildegard’s spiritual inspiration, regardless of whether the diagnosis might be correct. ‘Unlike Singer’, Newman writes, ‘we must avoid the reductionist error of assuming that a physiological cause (or better, correlative) of [Hildegard’s] visions excludes the possibility of any higher inspiration’.^68^ By contrast Sabina Flanagan took the opposite approach in her biography of Hildegard: Sacks had not gone far enough, she believed, in using Singer’s argument to linking the abbess’ illnesses to her works. Flanagan believed that it was possible to identify ‘each illness described by Hildegard as a manifestation of migraine’ and to correlate these experiences of illness with Hildegard’s production of visionary writings. Doing so allowed Flanagan to better understand how ‘the complex interaction of such physical and physiological factors’ had enabled Hildegard to assume her prophetic role. To this end, migraine experience had provided Hildegard with ‘a wonderfully adaptable instrument’.^69^

Newman responded to Flanagan by reiterating her position that although plausible, the migraine hypothesis was ‘excessively mechanical and reductive’. Newman emphasised that Hildegard’s own declarations about her chronic debilitating illnesses needed to be understood in their contemporary context, shaped by genuine and intense religious experience, the authority derived from human and feminine incapacity, as well as hagiographic conventions of the time. If, for Flanagan, the methodology of modern science was a historical resource, for Newman it was a red herring that Flanagan was using to protect Hildegard against accusations of ‘charlatanism’.^70^

Perhaps the strongest advocate for Hildegard’s migraine diagnosis has been the art historian Madeline Caviness, the first recent scholar to make a serious case for Hildegard’s role as designer of the *Scivias* miniatures. Caviness’ evidence includes stylistic conventions such as the depiction of drapery; the privileging of the feminine in several of the illuminations; the close correlation between text and imagery, as well as the transposition of ideas from Hildegard’s other writings; and the depiction of Hildegard with a wax tablet, on which, Caviness proposes, she may have sketched the designs.^71^ In addition to her robust art-historical methodology, however, Caviness has drawn on Singer’s migraine thesis as well as her own experiences of migraine aura, to support her argument that Hildegard is ‘surely as much the author of these pictorial ideas as she is of the words that she also did not physically write’. Caviness’ empathy as a fellow sufferer who recognises the distinctive jagged-edged and crenelated forms, black clouds and ‘tiny light points that make the contours shimmer’ is an important element of her authentication. The visual cues in the *Scivias* illuminations represent for Caviness ‘the most persuasive arguments for Hildegard’s close personal attention to the execution of the illuminations, since she was the one with migraine and knew these effects at first hand’.^72^ Medieval manuscripts were often produced by trained artists and scribes under instruction from authors. Even if Hildegard did not mix the paints or apply the brush for this ‘deluxe illuminated copy’ herself, Caviness argues, ‘the authentic rendition of these visual auras is thus best attributed to Hildegard herself (…) unless we suppose that an illuminator was found to work on the Rupertsberg *Scivias* who also had migraine’. Essentially, Caviness implies that *only* a migraineur could have made the images. For Caviness, establishing Hildegard’s role as designer of the images in *Scivias* (rather than the alternative view that they were produced later by professional artists, as recently suggested by Saurma-Jeltsch and Suzuki) is crucial because it constitutes ‘the last area of Hildegard’s multimedia outpourings that has been denied to her by recent scholars’.^73^

Caviness’ empathy with Hildegard as a ‘sufferer’ is not in question, neither is her expertise in bringing relevant art-historical evidence to her argument about the inseparability of image and text in *Scivias*. However, the use of Hildegard’s retrospectively diagnosed migraine does introduce a flaw to the argument; the historical uncertainties surrounding the creation of the *Scivias* manuscript miniatures, as well as the removal over time of Singer’s migraine theory from its original contexts lead Caviness into making a fallacy, or circular argument in this respect.^74^ If we first return to Charles Singer, we note that in order to make the argument that Hildegard suffered from migraine, Singer had to assume that Hildegard had a direct role in the production of the illuminations in *Scivias*. Singer’s own wording on this point changed subtly over time. While in the original Hildegard article (1917) he claimed that there was ‘strong evidence’ that the manuscript was either supervised by Hildegard herself, or ‘under her immediate tradition’, in the revised article (1928) this phrase became ‘little doubt’. As we have seen, by the time Sacks came to reproduce Singer’s argument in 1970, he entertained no doubt, and Hildegard became just one more medical case.^75^ In 1998, when Caviness applied the migraine argument in support of her claim that Hildegard was directly involved in the production of the *Scivias* miniatures, but the ‘medical’ evidence came with this integral initial *assumption* about Hildegard’s role in the illuminations firmly embedded. Independently, in their own disciplinary contexts, both the medical and historical elements of the argument are plausible, but they simply cannot be brought together without making a circular argument.^76^

It is perhaps unsurprising that Singer’s six-colour reproductions of the original *Scivias* illustrations, now lost, might suggest a tempting link to the ‘real’ Hildegard, but there is a further problem with adopting the migraine diagnosis as part of a feminist reclaiming of Hildegard and her canon. As we have seen, Singer’s original diagnosis of Hildegard served a particular purpose: it explained away her religion so that she could be fitted into a coherent account of medieval scientific progress. The evidence to advance Singer’s case was highly gendered because the inclusion of auras in medical explanations of migraine in the nineteenth century privileged the experiences of a small and elite group of scientific men. By diagnosing Hildegard with a ‘scientific’ migraine that negated her theology, a disorder explicitly coded male, Singer could fit her into his *longue durée* history of scientific vision, genius, objective reason and intellectual power. Caroline Walker Bynum has commented that ‘female saints are not canonised or revered unless they are in some way religiously useful to men’.^77^ In Hildegard’s case, this might also read ‘scientifically useful’. Quite rightly, historians such as Caviness are attempting to revise these assumptions, but in using the diagnosis of Hildegard’s migraine to do so, these projects subtly reaffirm, rather than move beyond, a gendered baggage of illness and authenticity that has worked to silence women’s voices and experiences, in this case of a common and debilitating disorder.^78^

## 7. Conclusion: Finding Meaning

In 2001, two Dutch physicians contributed an editorial comment to the leading headache journal *Cephalalgia*. Basing their comments on the visual appearance of some of Pablo Picasso’s artwork, they proposed that the artist had been a migraine sufferer.^79^ A decade later they admitted, with embarrassment, that ‘our suggestion that Picasso could have suffered from migraine auras without headache was, at that time, not based on research of biographies, letters or memoirs of Picasso or one of his contemporaries’.^80^ In the era of global instant media, their theory had spread, and their retraction was too late: a casual diagnosis released by medical ‘experts’ into the public realm had ensured that Picasso joined Hildegard as a famous sufferer of migraine. Does their absence of proof matter? In a recent memoir of living with migraine, Andrew Levy suggests that the reality of such a retrospective diagnosis is, in fact, irrelevant. For Levy, what matters is that

whatever their particular muses were, women and men like Hildegard and Picasso, like Woolf and Darwin, all went down the same deep well that the migraine sufferer reaches (…) their cases, taken together, tell us that what we call mystic vision, or creative inspiration, may be vastly more promiscuous and accessible than we generally allow, and that illness may be a gate swung wide open.^81^

For Levy, the importance of people like Hildegard and Picasso is their ability to translate the brain’s ‘elemental language’ into a meaningful form for people who recognise something of themselves in the story. In this context, the significance of these visionary experiences relates to positive creative possibilities that are removed from a need to believe in divine revelation, or connotations of negative pathology. From Charles Singer’s first presentations of his ideas about Hildegard in 1913, sufferers have recognised the *Scivias* illustrations as reflecting their own experiences of visual disturbance connected with migraine. One recent example of this meaning is on the internet, where contributors to forums discussed whether Hildegard would make a good patron saint of migraine. As one contributor comments: ‘I’m sure we can use all the saints we can get. I don’t think we found an “official” migraine saint. I didn’t check the archives but I think some of us just decided that Hildegard would be a good choice’.^82^ Whatever complicated historical contexts lie behind the life of Hildegard’s diagnosis, her continued popularity illustrates the problem with rejecting retrospective diagnosis outright. If we reject Singer’s diagnosis outright, we deny the real meaning that people *have* derived from recognising something of their own experiences in historical traces. On the other hand, it is likely that the narrow definition of migraine that the Hildegard imagery represents (a neurologically privileged definition that derived from the nineteenth century, and was revived in part by Oliver Sacks nearly a century later), excludes a large proportion of migraine sufferers who *do not* see their own experiences in these images, or who do not find in migraine an opportunity to release their creativity. How do we write histories that can take account of chronicity, nausea, periodicity and pain, rather than just visual disturbance?

Hildegard’s migraine shows the importance of paying attention to hidden assumptions that may be reproduced, or agendas served, however unwittingly, by reproducing out of context retrospective claims about the past. Historians need to examine the historical contexts that give rise to retrospective diagnoses, and to be aware that meanings or kinds of evidence may be silenced in the process. These diagnoses can take on a life of their own, and come to serve new purposes that we might later come to regret. The problem with retrospective diagnosis might not lie in its failure to respect the past, but the impossibility of predicting its future, although this, of course, could be said of any historical project.

## Footnotes

1. Charles Singer, ‘The scientific views and visions of Saint Hildegard (1098–1180)’ in C. Singer (ed.), *Studies in the History and Method of Science*(Oxford: Clarendon Press, 1917), 1–55: 51–5. Note that Singer was mistaken in his dates; Hildegard actually died in 1179.

2. For general works in English on Hildegard, see eg. Barbara Newman (ed.), *Voice of the Living Light: Hildegard of Bingen and Her World*(Berkeley, CA: University of California Press, 1998), 3–29; Sabina Flanagan, *Hildegard of Bingen: A Visionary Life*, 2nd edn (London and NY: Routledge, 1998); Charles Burnett and Peter Dronke (eds), *Hildegard of Bingen: The Context of Her Thought and Art* (Warburg Institute Colloquia, 4) (London: Warburg Institute, 1988).

3. For an excellent critique of interdisciplinary ‘borrowing’ see Constantina Papoulias and Felicity Callard, ‘Biology’s Gift: Interrogating the Turn to Affect’, *Body and Society*, 16, 1 (2010), 29–56.

4. AndrewCunningham, ‘Identifying Disease in the Past: Cutting the Gordian Knot’, Asclepio, 54, 1 (2002), 13–34: 17.

5. Laura J. McGough, ‘Syphilis in History: A Response to Two Articles’, *Clinical Infectious Diseases*, 41 (2005), 573–4; Roger Cooter, ‘The Life of a Disease?’, *The Lancet*, 375 (2010), 111–12.10.1086/43212716028179

6. For a review essay on disease ‘biographies’ see Anne Hardy, ‘A New Chapter in Medical History’, *Journal of Interdisciplinary History*, 39, 3 (2009), 349–59; Christopher Hamlin, *Cholera: the Biography*(Oxford: Oxford University Press, 2009); Sander L. Gilman, *Obesity: The Biography* (Oxford, Oxford University Press, 2010). Gilman has been an outspoken critic of retrospective diagnosis; see his review of Richard Schain, *The Legend of Nietzsche’s Syphilis* (Greenwood Press, 2001) in *Isis*, 93, 4 (2002), 733–4.

7. On the relationship between paleopathology and retrospective diagnosis see K.-H. Leven, “At times these ancient facts seem to lie before me like a patient on a hospital bed’ – Retrospective diagnosis and ancient medical history’ in Herman F.J. Horstmanshoff, Marten Stol and C.R. Van Tilburg (eds), *Magic and Rationality in Ancient Near Eastern and Graeco-Roman Medicine*(Leiden: Brill, 2004), 369–86.17152180

8. Bruno Latour, ‘On the partial existence of existing and nonexisting objects’, in Lorraine Daston (ed.), *Biographies of Scientific Objects*(Chicago, IL and London: University of Chicago Press, 2000), 247–69.

9. Lester K. Little, ‘Plague Historians in Lab Coats’, *Past and Present*, 213 (2011), 267–90; Vivien Nutton (ed.), *Pestilential Complexities: Understanding Medieval Plague*, Medical History Supplement No. 27 (London: University College London, 2008); Samuel K. Cohn, Jr, ‘The Black Death: End of a Paradigm’, *American Historical Review*, 107, 3 (2002), 703–38.

10. Monica Green, ‘The value of historical perspective’, in Ted Schrecker (ed.), *The Ashgate Research Companion to the Globalization of Health*(Farnham: Ashgate, 2012), 17–38: 24.

11. Caroline Walker Bynum, *Holy Feast and Holy Fast: The Significance of Food to Medieval Women*(Berkeley, CA, Los Angeles, CA and London: University of California Press, 1987), 194–218. In particular, Bynum was responding to Rudolph Bell’s *Holy Anorexia* (Chicago, IL: Chicago University Press, 1985), in which he identified ‘a historically significant group of women [who] exhibited an anorexic behaviour pattern in response to the patriarchal structures in which they were trapped’ (ix), and hoped to persuade physicians and psychologists to re-evaluate some aspects of modern approaches to the disease.

12. M.Lorch, ‘Language and Memory Disorder in the Case of Jonathan Swift: Considerations on Retrospective Diagnosis’, Brain, 129 (2006), 3127–31371702831010.1093/brain/awl246

13. It is particularly significant that the idea of Hildegard’s migraine spread before the ‘whirligig of the web’. See Angelique Richardson, ‘Thomas Hardy: Neither Boring nor Syphilitic’, *Critical Quarterly*, 50, 1–2 (2008), 234–9.

14. For recent discussions on the question of Hildegard’s authorship of *Causae et Curae* see Victoria Sweet, *Rooted in the Earth, Rooted in the Sky: Hildegard of Bingen and Premodern Medicine*, Studies in Medieval History and Culture, 19 (New York and London: Routledge, 2006), 35–49; Laurence Moulinier (ed.), *Hildegardis Bingensis Cause et Cure*, vol. 1 (Berlin: Rarissima Mediaevalia, 2003).

15. Translation from Latin in Priscilla Throop, *Hildegard of Bingen Causes and Cures*, 2nd edn (Charlotte Vermont: Medieval MS, 2008), 74–5, 135.

16. ‘Recette contre la migraine’ in Ernest Wickersheimer, ‘Textes Médicaux Chartrains Des IXe, Xe et Xie Siècles’ in E. Ashworth Underwood (ed.) *Science, Medicine and History: Essays on the Evolution of Scientific Thought and Medical Practice Written in Honour of Charles Singer*(London, New York & Toronto: Oxford University Press, 1953), 164–76. With thanks to Professor Anne Duggan for assistance in Latin translation.

17. John Pughe (trans.) and John Williams Ab Ithel, *The Physicians of Myddvai*(Llandovery: Longman & Co, 1861), 339.

18. ‘The life of holy Hildegard’, Book II: V & VIIII, in Anna Silvas (trans. & ed.), *Jutta and Hildegard: The Biographical Sources*(University Park, PA: Pennsylvania State University Press, 1999), 163, 169.

19. Charles Singer, ‘Autobiographical preface’ to *From Magic to Science*(New York: Dover, 1958), 8; Geoffrey Cantor, ‘Presidential Address Charles Singer and the Early Years of the British Society for the History of Science’, *British Journal of the History of Science*, 30 (1997), 5–23.11618883

20. Charles Ryell to Charles Singer, Wellcome Library, Singer Correspondence, PP/CJS/A.15 Correspondence R, 3 March 1911; Fielding H. Garrison to Charles Singer, WL Singer Correspondence, PP/CJS/A.7 Correspondence G, 6 October 1913.

21. Silvas, *op. cit.*(note 18), 159.

22. For overviews of Hildegard’s life and work see Peter Dronke, ‘Hildegard of Bingen’, in *Women Writers of the Middle Ages*(Cambridge and New York: Cambridge University Press, 1984), 144–201: 145; Barbara Newman, “‘Sybil of the Rhine”: Hildegard’s life and times’ in *Voice of the Living Light: Hildegard of Bingen and her World* (Berkeley, CA: University of California Press, 1998), 3–29; Sabina Flanagan, *Hildegard of Bingen: A Visionary Life*, 2nd edn (London and New York: Routledge, 1998); Kathryn Kerby-Fulton, ‘Hildegard of Bingen’, in Alastair Minnis and Rosalynn Voaden (eds), *Medieval Holy Women in the Christian Tradition c. 1100–1500* (Turnhout: Brepols, 2010), 343–69.

23. Newman, *ibid.*, 8–10.

24. One recent report suggests that three per cent of all Germany’s inhabitants express trust in ‘Hildegard Medicine’, a practice the authors warn is the highly commercialised product of two physicians (Gottfried Hertzka and Wighard Strehlow) and based on ‘poorly defined overpriced preparations’. See O. Micke and J. Hubner, ‘Traditional European Medicine – After All, is Hildegard von Bingen really right?’, *European Journal of Integrative Medicine*, 1 (2009), 226; *Vision* Dir. Margarethe von Trotta (2009).

25. Newman, *op. cit.*(note 22), 1.

26. Monica Green, ‘In Search of an “Authentic” Women’s Medicine: The Strange Fates of Trota of Salerno and Hildegard of Bingen’, *Dynamis*, 19 (1999), 25–54: 51; Florence Eliza Glaze, ‘Medical writer’, in Newman, *op. cit.*(note 22), 125–48.11624264

27. Sweet, *op. cite.*(note 14), 6.

28. Mélanie Lepinska, *Histoire des Femmes Médecins depuis l’Antiquité jusqu’a nos jours*(Paris: Librairie C. Jacques & Companie, 1900), 133.

29. Singer, *op. cit.*(note 19), viii.

30. Arnold Klebs to Singer 29 July 1913, WL PP/CJS.A.10 Correspondence K. It is likely that Klebs is referring to photographs that Louis Baillet lent Singer, as Singer credited in the Hildegard chapter.

31. ‘St Hildegard’, *Proceedings of the Royal Society of Medicine*(History of Medicine Section), vii (1913), 1–2; ‘Medical Societies’, *Transactions of the Royal Society of Medicine: Section of History of Medicine* (1913), 1540.

32. Singer, *op. cit.*(note 19), ix.

33. Singer, *op. cit.*(note 1), 52–3. Singer quoted from Jacques-Paul Migne (ed.), *Patrologia Latina*, vol. 197 (Paris: Migne, 1855).

34. John Herschel, ‘On sensorial vision’, in *Familiar Lectures on Scientific Subjects*(New York and London: George Routledge and Sons, 1872), 400–18: 408–9. Herschel also made records in his diary eg. ‘a most superb and perfect *Turkshead pattern* (…) as sharp as if drawn with compasses’ in entry for 18 August 1855, Royal Society of London MS 584.

35. Sir David Brewster, ‘On Hemiopsy, or Half-Vision’, *The London, Edinburgh and Dublin Philosophical Magazine and Journal of Science*, XXIX, 4 (1865), 503–7; G. B. Airy ‘On hemiopsy’, *The London, Edinburgh, and Dublin Philosophical Magazine and Journal of Science*, 30 (1865), 19–21.

36. Elizabeth Green Musselman, *Nervous Conditions: Science and the Body Politic in Early Industrial Britain*(Albany: State University of New York, 2006), see especially Chapter 4, ‘Mental governance and hemiopsy’, 101–45.

37. Hubert Airy, ‘On a distinct form of transient hemiopsia’, *Philosophical Transactions of the Royal Society*, 160 (1870), 247–64: 255, 264.

38. Apart from his admission of headache, Airy made one passing reference in a footnote to a mention of visual aura in Fothergill’s 1778 ‘Remarks on Sick Headach’, which had been ‘very lately’ drawn to his attention; Airy, *ibid.*, 254.

39. Edward Liveing, *On Megrim, Sick-Headache and Some Allied Disorders*(J. & A. Churchill: London, 1873).

40. Peter Wallwork Latham, *On Nervous or Sick-Headache, its Varieties and Treatment*(Cambridge: Deighton, Bell and Co; London: Bell and Daldy, 1873), 16. For a study of Latham and Liveing, see Mark Weatherall, ‘The Migraine Theories of Liveing and Latham: A Reappraisal’ *Brain*, 135, 8 (2012), 2560–8.10.1093/brain/aws02022382360

41. Latham, *ibid.*, 2; Liveing, *op. cit.*(note 39), 25–7.

42. See for instance, P.J. Murphy, ‘On Headache and Its Varieties’, *The Lancet*, 63, 1590 (1854), 182–3.

43. See for example, Alex W. Stirling, ‘On Certain Subjective Visual Sensations’, *Journal of the American Medical Association*, 27, 23 (1896), 1181–4; Alexander Wallace, ‘On Migraine’, *The Lancet*, 141, 3620 (1893), 79–81; W. Pemberton Peake, ‘A Few Observations on the Pathology and Treatment of Migraine’, *The Lancet*, 136, 3500 (1890), 666.

44. M.J. Eadie, ‘Hubert Airy, Contemporary Men of Science and the Migraine Aura’, *Journal of the Royal College of Physicians of Edinburgh*, 39, 3 (2009), 263–7: 267.20608346

45. WilliamGowers, ‘Subjective Visual Sensations’, Transactions of the Ophthalmological Society of the United Kingdom, 15 (1895), 1–38, 25.

46. Lorraine Daston and Peter Galison, *Objectivity*(Zone Books: New York, 2010), 19–20.

47. Jean-Martin Charcot, *Clinical Lectures on Diseases of the Nervous System [1882–5]*, trans. Thomas Savill [1889], 62–8.

48. Amanda Power, ‘A Mirror for Every Age: The Reputation of Roger Bacon’, *English Historical Review*, 121, 492 (2006), 657–92, 676–7; Andrew Cunningham has also critiqued recent attempts to create ‘saint-scientists’ in ‘Science and Religion in the Thirteenth Century Revisited: The Making of St Francis the Proto-Ecologist. Part 1: Creature not Nature’, *Studies in History and Philosophy of Science*, 31, 4 (2000), 613–43.

49. Singer, ‘The Development of the Doctrine of Contagium Vivium, 1500–1750’ (London: privately printed, August 1913), 12. WL MS PP/CJS B.1: Writings by Charles Singer, 1905–14.

50. Anna K. Mayer, ‘When Things don’t Talk: Knowledge and Belief in the Inter-War Humanism of Charles Singer’, *British Journal for the History of Science*, 38, 3 (2005), 325–47: 326–7.10.1017/s000708740500700416240547

51. Singer, *op. cit.*(note 1), 30–2.

52. Charles Singer, ‘Allegorical Representation of the Synagogue in a Twelfth Century Illuminated Manuscript of Hildegard of Bingen’, *The Jewish Quarterly Review*, New Series 5, 8 (1915), 267–88: 269, n. 8; Singer, *op. cit.*(note 1), 12–13. For recent discussions about Hildegard’s authorship of the medical texts known as *Physica* and *Causae et Curae* see Green, *op. cit.* (note 26), 45; Faith Wallis (ed.), *Medieval Medicine: A Reader* (Toronto, ON: University of Toronto Press, 2010), 357–8.

53. Charles Singer, ‘Science’, in F.J.C. Hearnshaw, *Mediaeval Contributions to Modern Civilization*(London, 1921), 106–48: 111; Charles Singer, ‘What is Science?’, *British Medical Journal*, 1 (1921), 954.

54. Singer, *op. cit.*(note 19), xii–xiii.

55. R.F.Elliott, ‘Migraine and Mysticism’, Postgraduate Medical Journal, 8, 86 (1932), 449–4592131277310.1136/pgmj.8.86.449PMC2532152

56. Macdonald Critchley and Fergus R. Ferguson, ‘Migraine’, *The Lancet*, 1 (1933), 182–5, and *The Lancet*, 1 (1933), 123–5.

57. ‘Dr Sacks on Migraine’, http://www.oliversacks.com/books/migraine/ (accessed 14 May 2013).

58. Oliver Sacks, *Migraine: Understanding the Common Disorder*(London: Pan Books, 1985), 106–8. All references to *Migraine* hereafter are to this edition unless otherwise stated.

59. Sacks, *ibid.*, 55.

60. M.J.Eadie, ‘The Pathogenesis of Migraine – 17th to Early 20th Century Understandings’, Journal of Clinical Neuroscience, 12, 4 (2005), 383–3881592576710.1016/j.jocn.2004.12.003

61. Sacks, *op. cit.*(note 58), 192.

62. Peter J. Goadsby, ‘The Vascular Theory of Migraine – A Great Story Wrecked by the Facts’, *Brain*, 132, 1 (2009), 6–7; for a response to Goadsby that is little short of furious see Elliott Shevel, ‘The Extracranial Vascular Theory of Migraine: an Artificial Controversy’, *Journal of Neurological Transmission*, 118 (2011), 525–30.10.1007/s00702-010-0517-121207080

63. See eg. Oliver Sacks, *Migraine*(Berkeley, CA: University of California Press, 1985, 1986 & 1993).

64. Subtitles to *Migraine*include *Evolution of a Common Disorder*(1970–73); *Understanding a Common Disorder* (1985–87). By the 1990s, the subtitle had been dropped in favour of ‘Revised and Expanded’.

65. Oliver Sacks, *Migraine*(Berkeley, CA: University of California Press, 1993), xv.

66. Sally Shuttleworth, *The Mind of the Child: Child Development in Literature, Science and Medicine, 1840–1900*(Oxford: Oxford University Press, 2010), 22.

67. Esther Cohen, *The Modulated Scream: Pain in Late Medieval Culture*(Chicago, IL and London: University of Chicago Press, 2010), 118; Joseph L. Baird and Radd K. Ehrman, Hildegard of Bingen to Christian, Archbishop Of Mainz, 1179, in *The Letters of Hildegard of Bingen*, vol. 1 (Oxford: Oxford University Press, 1994), 80–2.

68. Barbara Newman, ‘Hildegard of Bingen: Visions and Validation’, *Church History*, 54, 2 (1985), 163–75: 167.

69. Sabina Flanagan, *Hildegard of Bingen, 1098–1179: A Visionary Life*, 2nd edn (London and New York: Routledge, 1998), 191–202: 200.

70. Barbara Newman, ‘Three-part invention: the Vita S. Hildegardis and mystical hagiography’, in Charles Burnett (ed.), *Hildegard of Bingen: The Context of her Thought and Art*(London: The Warburg Institute, 1998), 189–210: 197–8.

71. This argument is explored most fully in Madeline Caviness, ‘Gender symbolism and text image relationships: Hildegard of Bingen’s Scivias’, in Madeline Caviness (ed.), *Art in the Medieval West and its Audience*(Aldershot: Ashgate, 2001), 71–108. Previously, some authors had simply assumed Hildegard’s role. See eg. Matthew Fox, *Illuminations of Hildegard of Bingen* (Rochester: Bear & Co: 1985).

72. Madeline Caviness, ‘Hildegard as designer of illustrations’, in Charles Burnett and Peter Dronke (eds), *Hildegard of Bingen: The Context of Her Thought and Art*(London: Warburg Institute, 1998), 29–62: 33.

73. Madeline Caviness, ‘Artist: “To See, Hear, and Know All at Once”’, in Newman (ed.), *Voice of the Living Light,*110–124: 110, 113; Lieselotte E. Saurma-Jeltsch, *Die Miniaturen im “Liber Scivias” der Hildegard von Bingen: Die Wucht der Vision und die Ordnung der Bilder*(Wiesbaden: Dr Ludwig Reichert, 1998); Keiko Suzuki, Bildgewordene Visionen oder Visionserzaihlungen (Bern: Peter Lang, 1998). For Caviness’ critique of these two works see Madeline H. Caviness, ‘Hildegard of Bingen: Some Recent Books’, *Speculum*, 77, 1 (2002), 113–120: 119.

74. Vivian Nutton has warned of the need to ‘take the utmost care not to proceed along a path that is ultimately circular’. ‘Introduction’ to V. Nutton (ed.), *Pestilential Complexities: Understanding Medieval Plague*(London: The Wellcome Centre Trust for the History of Medicine, 2008), 11, 16.

75. Compare Singer, ‘Scientific Views and Visions [1917]’, 7 with Singer, ‘Scientific Views and Visions [1928]’, 205. In 1913, the Royal Society of Medicine Meeting report used the phrase ‘almost certainly prepared under Hildegard’s personal supervision’, although it is unclear whether these are Singer’s words.

76. Charles Singer, ‘Preface’ to *From Magic to Science: Essays on the Scientific Twilight*(London: Ernest Benn, 1928), xii. In the 1928 version, Singer had also removed the highly criticised sections from the article in which he rejected Hildegard’s authorship of the medical manuscript *Causes and Cures* Singer’s reservations about the quality of *Causae et Curae* in comparison with her other works are understandable; debates about manuscript authenticity and the authorship of *Causae et Curae* (whether, for example, it was Hildegard’s work, but a rough draft) continues to be debated. However, while not integral to his discussion about migraine which refers only to *Scivias*, Singer’s removal of the discussion about *Causae et Curae* further removed of any sense of doubt in the piece, allowing historians who have only consulted the 1928 or 1958 versions of his article to forget that debates about manuscript authenticity were as contested in Singer’s time as they are today.

77. CarolineWalker Bynum, Fragmentation and Redemption: Essays on Gender and the Human Body in Medieaval Religion (New York: Zone, 1991), 17

78. See eg. G.D. Schott, ‘Exploring the Visual Hallucinations of Migraine Aura: The Tacit Contribution of Illustration’, *Brain*, 130 (2007), 1690–703: 1694.10.1093/brain/awl34817264093

79. M.D.Ferrari and J. Haan, ‘Migraine Aura, Illusory Vertical Splitting, and Picasso’, Cephalalgia, 20, 8 (2000), 6861116789510.1111/j.1468-2982.2000.00113.x

80. JoostHaan and Michel D. Ferrari, ‘‘Picasso’s Migraine: Illusory Cubist Splitting or Illusion?’’, Cephalalgia, 31, 9 (2011), 1057–10602157175810.1177/0333102411406752

81. AndrewLevy, A Brain Wider than the Sky: A Migraine Diary (New York: Simon and Schuster, 2009), 104

82. See http://www.mombu.com/medicine/medicine/t-hildegard-of-bingen-migraine-4798218.html (accessed 14 May 2013).

